# CODA (crossover distribution analyzer): quantitative characterization of crossover position patterns along chromosomes

**DOI:** 10.1186/1471-2105-12-27

**Published:** 2011-01-20

**Authors:** Franck Gauthier, Olivier C Martin, Matthieu Falque

**Affiliations:** 1 UMR de Génétique Végétale, INRA - Univ Paris-Sud - CNRS - AgroParisTech, Ferme du Moulon, F-91190 Gif-sur-Yvette, France

## Abstract

**Background:**

During meiosis, homologous chromosomes exchange segments via the formation of crossovers. This phenomenon is highly regulated; in particular, crossovers are distributed heterogeneously along the physical map and rarely arise in close proximity, a property referred to as "interference". Crossover positions form patterns that give clues about how crossovers are formed. In several organisms including yeast, tomato, *Arabidopsis*, and mouse, it is believed that crossovers form via at least two pathways, one interfering, the other not.

**Results:**

We have developed a software package - "CODA", for CrossOver Distribution Analyzer - which allows one to quantitatively characterize crossover patterns by fitting interference models to experimental data. Two families of interfering models are provided: the "gamma" model and the "beam-film" model. The user can specify single or two-pathways modeling, and the software package infers the model's parameters and their confidence intervals. CODA can handle data produced from measurements on bivalents or gametes, in the form of continuous crossover positions or marker genotyping. We illustrate the possibilities on data from Wheat, corn and mouse.

**Conclusions:**

CODA extends the kind of crossover data that could be analyzed so far to include gametic data (rather than only bivalents/tetrads) when using two-pathways modeling. It will also enable users to perform analyses based on the beam-film model. CODA implements that model's complex physics and mathematics, and uses a summary statistic to overcomes the lack of a computable likelihood which has hampered its use till now.

## Background

In sexually reproducing organisms, haploid gametes are produced during meiosis. Fertilization then restores the diploid number of chromosomes via the fusion of two gametes to form a zygote. A major consequence of meiosis is that the genetic material of the parents undergoes two levels of shuffling: (1) the chromosomes segregate independently, and (2) intra-chromosomal recombination occurs through reciprocal exchanges of chromosome segments due to crossing-over between homologs during prophase I of meiosis. Crossovers (COs) thus drive genetic diversity through recombination, and they are also essential for proper chromosome segregation because they hold homologous chromosomes together at metaphase I [1]. The genetic distance between two loci is defined as the average number of COs within this interval, per meiosis, when considering gametes. Thus a 100 cM (1 Morgan) segment has on average 1 CO per gamete, and 2 COs per bivalent (the bivalent including two homologous chromosomes during the first meiotic division). In most organisms, CO formation is highly regulated with respect to the number and the distribution of CO events on the chromosomes. This distribution is clearly non-random; some regions of the physical chromosome are much more prone to CO formation and recombination than others [2].

Furthermore, a phenomenon called CO interference [3] lowers the probability that two COs occur close to each other in the same meiosis. Recent evidence highlights that COs form within two different pathways: the interfering (hereafter referred to as P1) and the non-interfering (hereafter referred to as P2) pathways. The proportion *p *of COs formed through P2 is quite variable. This proportion has been estimated in different species via biological [4,5] or computational [6-8] approaches, with typical values between 5 and 30%.

At present there is no efficient tool for targeting COs to desired chromosomal locations in higher plants. Since breeding relies on selecting progenies where COs have assembled together favorable alleles that were separated in the parental genotypes, getting insights into the mechanisms of CO formation and characterizing the number and localization of COs is of key importance. A first characterization of CO distributions is achieved by extracting (i) the strength of interference at work in P1 and (ii) the proportions of P1 and P2 COs.

To characterize interference strength, the most powerful approach is to fit mathematical models to experimental data sets. Such models may be grouped into two classes: physically motivated models and statistically oriented models. The main physical model is the *beam-film *(BF) model [9]. It considers the establishment and propagation of a mechanical stress using a mechanical analogy of a ceramic film on a metallic beam, with COs being seen as "cracks" which release the stress locally in the film and thus forbid nearby COs. The statistical models are mainly based on the statistics of genetic distances between successive COs, using stationary renewal processes (SRPs), and no chromatid interference [10,11]. Among SRP-based models, one of the most studied is the *gamma *model, which generates inter-CO distances using the gamma distribution [10]. To include a non-interfering pathway, non-interfering COs are simply added to those of the interfering pathway, leading to the gamma "sprinkling" (GS) [6-8] and BF sprinkling (BFS) [8] models.

Fitting these kinds of models to data requires rather complex mathematics and computer programming. It has thus long been difficult for data producers to analyze their CO distributions without collaborating with specialized groups. To try to fill this gap, Viswanath & Housworth [12] developed a Java application based on two pathways modeling where P1 is described by the counting model (a particular case of the *gamma *model). However this software tool is restricted to marker segregation data *in tetrads*, a data type that is available only for particular organisms like Ascomycetes or for one particular *Arabidobsis *mutant. It is thus not applicable to the vast majority of organisms, for which marker segregation data are obtained from individual gametes (or their progeny), rather than from tetrads or bivalents. More recently, Housworth & Stahl [13] published a R script to estimate interference strength from CO position data using the single pathway *gamma *model. Unfortunately, the script does not allow two-pathways analyses: this compromises realistic descriptions of CO distributions whenever two pathways are at work, which is the case in the vast majority of species studied so far. Moreover, this R script was designed to analyze positions of protein foci immunolocalized on synaptonemal complexes, which is equivalent to positions of P1 COs on the *bivalent*. So here as well as with the previous software, it is not possible to analyze marker segregation data in gametes or genetic mapping populations; this is all the more a drawback that those are the most common data sets available from plants and animals.

To our knowledge, no tool has been available to perform two-pathways analyses of CO distributions on segregation data obtained from individual gametes, for instance through linkage mapping experiments. Moreover, we do not know of any software for using physically motivated models like the BF model to analyze CO distributions. This last point is important because model-based inferences may often be considered suspiciously if their robustness to different model choices cannot be evaluated. So having predictions from two very different models gives further credence to these kinds of analyses and to their inferred parameters.

## Implementation

This software allows one to analyze CO position data sets or genetic mapping data to quantitatively characterize CO distributions along chromosomes. CODA works with two different types of datasets: (1) marker segregation data at the gamete level, obtained for instance from backcross or double-haploid linkage mapping populations, or from sperm typing, or (2) continuous CO positions such as obtained from immunolocalization of protein foci on synaptonemal complexes, corresponding to bivalents. These positions can be given in whatever units, including micrometers of synaptonemal complex or Mb of DNA sequence. CODA converts all of these into genetic positions to perform the fits in this space.

Two different interference models are implemented: the statistical gamma model and the physical BF model. When using the gamma model (either in a single or two-pathways framework), the adjustment of parameters is done using maximum likelihood [6,10]. In the case of the BF model, no likelihood can be computed, so we have developed a score based on a projected likelihood to measure the goodness of fit [8]. This score quantifies the differences between histograms produced from the experimental data and predicted by the model with its parameters; these histograms are for both inter-CO distances and numbers of COs per chromosome. The user can compare scores for different models to see which fits best. CODA provides two methods for determining the optimum model parameters: either by scanning the parameter space via a grid, or by applying hill-climbing -- performing small steps in the two-dimensional parameter space towards better goodness-of-fits, until no local improvement is found. In terms of speed, both methods have comparable efficiencies for single-pathway models, but with two pathways, the hill-climbing is much more effective and is strongly recommended for reasonable computation times. In terms of reliability, the hill-climbing may be affected by local maxima, but in all of our tests with real CO data, such a situation did not arise if the size of the population simulated was 10^6 ^or more. In case of doubt, users may prefer to use the scanning method, which is insensitive to local maxima.

Using the scanning algorithm, the likelihood or score can be displayed as a 3 D surface plot, whereas using the hill-climbing algorithm, CODA provides on the fly a graph of the trajectory in the space spanned by *parameter *1 and *parameter *2. All these aspects will be illustrated with figures in the "Results" section. CODA may be used in command-line mode to compute confidence intervals based on re-simulations, as was done in [8]. However, since that approach requires substantial CPU resources, the CODA GUI provides confidence intervals for the gamma model based on Fisher's information matrix.

## Software architecture

The core of CODA (*i.e*., the computing part) is written in standard C/C++, while the CODA graphical user interface (GUI) uses cross-platform C++ Qt libraries, and Qt based Qwt and Qwtplot3 d libraries for specialized 2 D and 3 D (respectively) plotting widgets.

Qt, Qwt and Qwtplot3 d are distributed under the terms of the GNU Lesser General Public License.

## Results

The result of running CODA is an estimation of interference strength (*parameter 1*), and in the case of two-pathways models, an estimate of the proportion of COs coming from each pathway (*parameter 2*). In addition, the user interface provides three characteristic graphs displaying features of the experimental CO patterns, as well as a visual comparison between models and experimental data during the adjustment process. Upon completion of analysis, one may export graphs as bitmap or vector images.

The GUI allows the user to choose the search method for determining the optimum model parameters: either by scanning the parameter space with a grid, or by performing hill-climbing in the goodness-of-fit.

We first present how to use the GUI to analyze a specific dataset (using the Wheat chromosome IIIB segregation data of [14]). Then we compare the outcomes of analyses (using mouse genetic mapping data of [15]) when employing (1) the gamma-sprinkling *vs *the beam-film sprinkling model; (2) the single *vs *two-pathways gamma model. Finally, we benchmark the speed of our software to that of existing tools on yet another data set (namely Maize late nodules data in electron microscopy from [16]).

### Graphical User Interface

#### Description of the "Settings" tab

**Figure 1:**
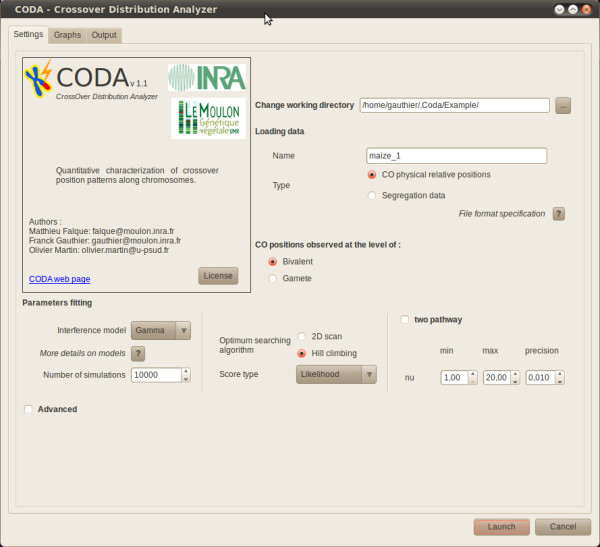
**Screenshot of the "Settings" tab of the GUI**. See text for explanations of the different items.

Figure 1 shows the interface for this tab, which provides the different items for specifying the desired analysis. These include the model type, the number of simulations, the search algorithm to find optimal parameters, the score type, and the parameter range to consider. Parameters here are the interference intensity and the proportion of non-interfering COs. The user can impose that the model considers one or two pathways.

#### Description of the "Graphs" tab

The upper part of this tab provides three types of graphs, the lower part displays current and best parameters values, as well as the confidence intervals for each parameter (gamma-based model only). Both parts are real-time updated throughout the duration of the fit. At any time, one can switch from one graph to another, keeping the parameters panel visible.

**Figure 2:**
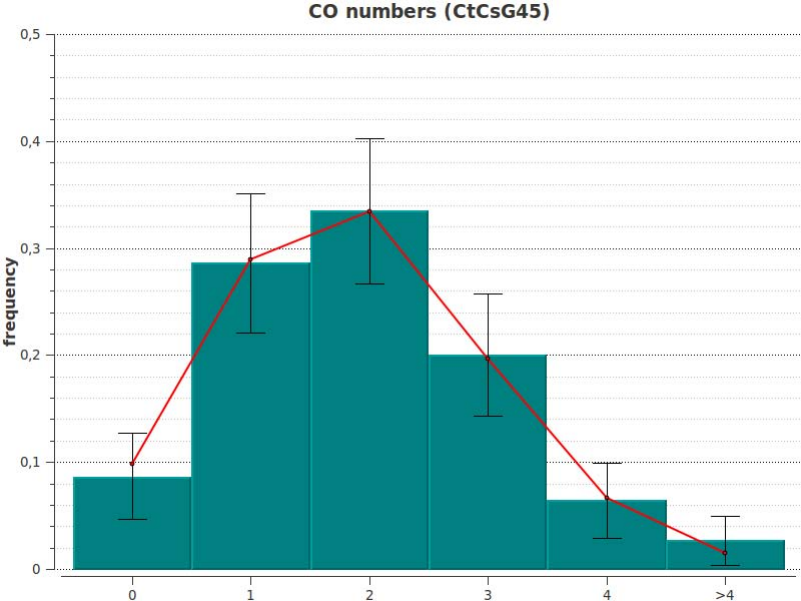
**Histogram showing the distribution of the number of COs**. Graph generated by CODA. Experimental data set (green with bars for 95% confidence intervals) is taken from Wheat chromosome IIIB segregation data [14]. Simulated data (red curve) were generated by the two-pathways gamma-sprinkling model.

**Figure 3:**
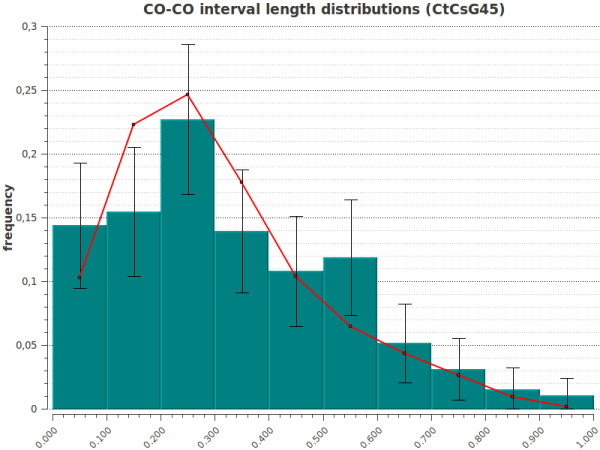
**Histogram showing the distribution of distances between successive COs**. Graph generated by CODA. Experimental data set (green with bars for 95% confidence intervals) is taken from Wheat chromosome IIIB segregation data [14]. Simulated data (red curve) were generated by the two-pathways gamma-sprinkling model. The *x *axis shows the relative genetic distance.

CODA produces several graphs. (i) The hill-climbing trajectory graph (if the hill-climbing algorithm was selected) shows the trajectory (in the parameters space) from the starting point (initial parameter values) to the peak of the likelihood (or score) function which corresponds to the best parameter values. (ii) The likelihood (or score) surface plot (if the 2 D scan algorithm was selected) is a 3 D visualization of the score/likelihood *vs *the parameters. The user can easily manipulate the 3 D view using the mouse and/or keyboard, to rotate, translate vertically or horizontally, zoom in/out, or stretch/compress the *z *axis (likelihood/score). (iii) Histograms give the possibility to graphically visualize agreement between simulated data generated by the model (red curves) and experimental data (green histogram bars) on the same plot. For the experimental data, the 95% confidence intervals are also displayed. The three categories of histograms available are: (1) The distribution of the number of COs per chromosome. Figure 2 gives an example of such graphical output when using genetic segregation data of Wheat chromosome IIIB, (2) the distribution of the CO positions along the chromosome, and (3) the distribution of *distances *between successive COs. Figure 3 gives an example of such graphical output when using genetic segregation data of Wheat chromosome IIIB. In this example, the discrepancy in the second bin between experimental and simulated values suggests that there is still room for improvement in today's interference models.

Bitmap (png, jpeg, bmp) or vector (pdf, eps, ps, svg) images of all graph can be generated.

### Comparing different models

#### Gamma sprinkling *vs *Beam-film sprinkling

**Figure 4:**
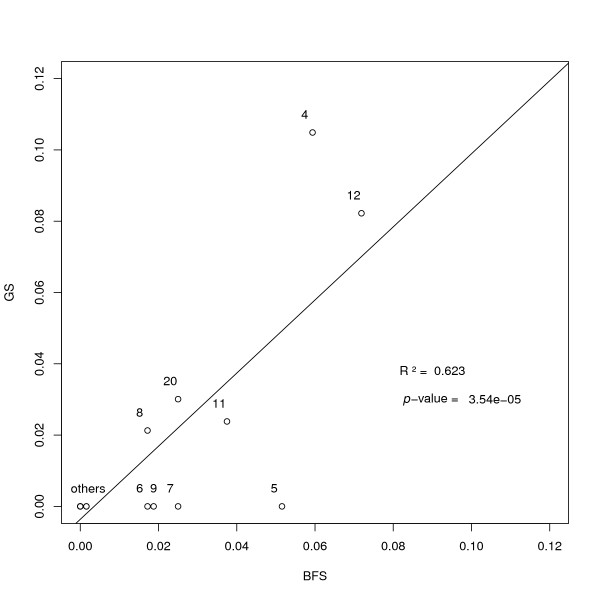
**Comparison of inferred values for the parameter *p *when using the gamma sprinkling (*y *axis) and the beam-film sprinkling models (*x *axis)**. Data is for the 20 autosomal chromosomes of mouse [15]. The regression line is shown on the graph.

Although the gamma and beam-film models are very different in spirit, the incorporation of sprinkling (pathway P2) has exactly the same meaning in both. It is thus appropriate to compare each model's prediction for the fraction *p *of crossovers coming from the non-interfering pathway P2. To do so, we analyzed mouse backcross data [15] using CODA. In Figure 4 we display the values of *p *inferred within each model (BFS and GS), for all 20 autosomal chromosomes. If the data sets were sufficiently large, one could expect the models to agree reasonably well, leading to a figure with clustering along the diagonal. In practice we see a bit of that but here the data set is not very large (less than 200 gametes); more convincing results were observed in a Maize data set which had greater statistical power [8]. In general, if two rather different models lead to similar estimates for *p*, one can argue that systematic biases due to the modeling are modest; such a check for robustness of predictions gives added weight to the analysis. In the present example, in spite of the low statistics, we also see that the beam-film model tends to predict larger values of *p *than the gamma model; a similar effect was seen in an analysis of the two pathways in Maize [8].

#### One *vs *two pathways modeling

**Figure 5:**
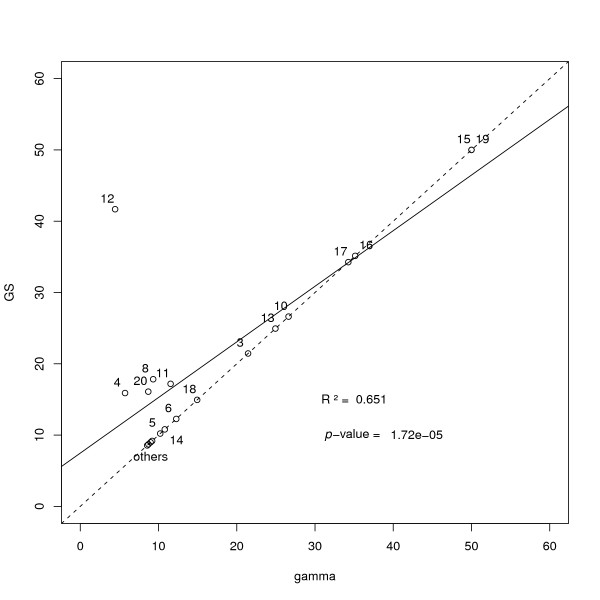
**Comparison of inferred values for the parameter *nu *when using the gamma sprinkling (*y *axis) and the single pathway gamma models (*x *axis)**. Data is for the 20 autosomal chromosomes of mouse [15]. Solid line: regression line. Dashed line: the first diagonal. For the chromosomes aligned on the first diagonal, the value of parameter *p *was estimated to zero.

Only in the last few years has it been realized that crossovers form via at least two pathways. Given the further complexity this adds to modeling, many groups analyzing crossover data tend to stay within the single pathway framework. Such a choice certainly affects the estimates of the interference strength. To examine this issue, we analyze here the mouse data of [15] using the gamma model of interference, allowing or not sprinkling, *i.e*., a second pathway. Presumably, forcing the presence of just one pathway systematically underestimates the values of the parameter *nu*. Our analyses of the 20 autosomes in mouse are displayed in Figure 5. For those chromosomes where GS estimates *p *to be zero, the predicted value of *nu *is of course the same in the two analyses. For the other chromosomes, we see as expected that the single pathway modeling systematically underestimates the value of *nu*.

### Performance benchmarks (computing time)

To quantify computational performances, we have used the platform GNU/Linux Ubuntu 10.04 32 bit, RAM 4 Go, 4 × Intel Xeon CPU E5410 2.33 GHz.

#### Comparison of fitting algorithms in CODA

In our first benchmark, we compare the computing times when using the *hill-climbing vs the complete scan *method for fitting the model's parameters. The analysis presented here is for Maize late nodule data [8], chromosome 1, using the *gamma sprinkling *model, for which we have crossover positions on bivalents, a situation relevant for the upcoming benchmark.

Table 1 gives the associated CPU times as well as the inferred parameters. We see that hill climbing is much faster. *N.B*.: if *step *is halved, the scan time is multiplied by 4, whereas the hill-climbing computing time is relatively insensitive to *precision*.

**Table 1:** Benchmark comparison between algorithms "complete scan" and "hill-climbing"

	Computing time	Interference strength (*nu*)	*p*
Complete scan	7 h 10 min	5.60	0.180

Hill-climbing	2 min 40 s	5.72	0.185

Complete *scan *results obtained with the GS model using the likelihood, for 1 ≤ nu ≤ 15 and a *step *of 0.05; 0 ≤ p ≤ 0.5 and a *step *of 0.01. *Hill-*climbing results were obtained with a *precision *of 0.01 and 0.001 for *nu *and *p *respectively. Data is Maize late nodule positions on chromosome 1 [8].

#### Existing tools comparison: CODA *vs *Interference Analyzer

In our second benchmark, we compare CODA to the software « Interference Analyzer » (IA) [12]. IA is the only other software we are aware of which allows the user to perform two pathway analyses, but it is restricted to bivalent or tetrad data (it does not handle "thinning", *i.e*., the loss of information on COs arising when one has data only from individual gametes or segregating populations). Shown in Table 2 are the computing times required by IA and CODA. The data is the same as for the previous benchmark, coming from Maize late nodules (giving CO positions along a bivalent, and thus analyzable using IA). The IA software works with the counting model [17] which restricts the parameter *nu *to integer values; this explains the slightly different fitted values for the two softwares. We see from this benchmarking that CODA is faster. Furthermore, CODA is able to treat additional data types such as generated by genetic mapping experiments on gametes; unfortunately, IA cannot handle such data types.

**Table 2:** Benchmark comparison between CODA and Interference Analyzer (IA)

	Computing time	Interference strength	*p*
IA	35 min	5	0.190

CODA	2 min 40 s	5.720	0.185

Comparison of CPU times required by IA and CODA. Data is Maize late nodule positions on chromosome 1 [8].

## Discussion

CODA provides both a quantitative and qualitative advance for the analysis of crossover data. With its ability to treat two pathway models and data coming from gametes, along with its easy use thanks to a GUI, CODA will allow researchers to perform their own analysis quite straightforwardly. It also allows users to compare the relative merits of the gamma model and the BF model.

Furthermore, the software package is evolvable: because it is open source, the users can modify the details of the models implemented, or even substitute their own choice of models. With data sets of crossover patterns growing in size and number, the number of potential users will increase. One can also expect that researchers will request more sophisticated models; we thus anticipate that we will be upgrading CODA to include these new models from which their added value can be tested.

**Figure 6:**
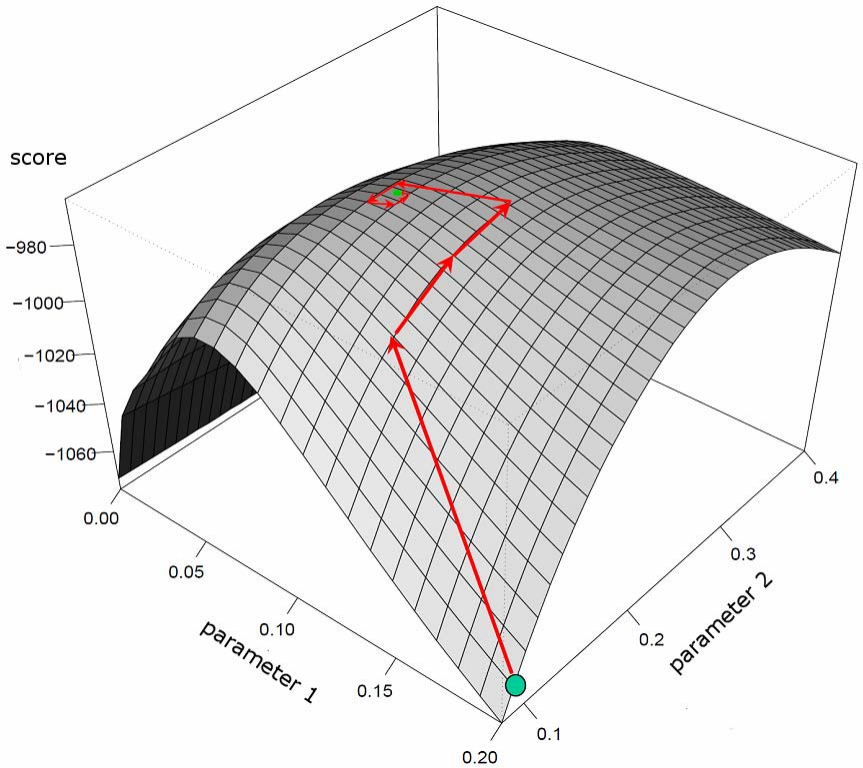
**Surface plot showing the *score *(*z *axis) as a function of interference strength (*parameter *1), and fraction of COs coming from pathway 2 (*parameter *2)**. The experimental data set is taken from Maize chromosome 1 late recombination nodules [8]. *Score *is for the two-pathways BF-sprinkling model. Superposed on this surface plot is a trajectory generated using the *hill-climbing *algorithm, indicating that this approach is very efficient because the hill to be climbed is smooth and single-peaked.

Given these different uses of CODA, it is appropriate to bring up the following technical point. We showed in the previous section that searching for the optimum parameters via scans is computationally slow. Nevertheless, for the hill climbing to work properly, it is important that there be a single peak (local maximum) in the hill to be climbed; verifying that this is the case typically requires a scan, albeit a crude one. In all the cases we have investigated, the hill landscape has been smooth with a single peak. Figure 6 shows an example in Maize which illustrates the associated efficiency of hill climbing in such a situation. If possible, the user should check this property on his particular data set, and an in depth study will be required if the user implements other models of his making.

## Conclusions

The open-access utility CODA provides the user with an easy interface for model-based characterizations of crossover patterns along chromosomes. It allows one to estimate the strength of crossover interference, using either the statistically motivated *gamma *model, or the mechanically formulated *beam-film *model. It can also be used for two-pathways modeling, where a second (non-interfering) pathway is superposed on the first, and generates multiple histograms that summarize the features of crossover patterns. The experimental input files can contain marker segregation data coming from genetic linkage mapping experiments, or crossover positions on chromosomes coming for instance from cytological imaging, significantly extending the possibilities of previous software packages. The use of this kind of modeling can give support to the presence of putative pathways as was done in [8]. Also, as the mechanisms of crossover formation become better known, more sophisticated models can be added to CODA to exhibit their characteristics and to quantify their level of agreement with experiments, thereby advancing the detailed understanding of meiotic processes.

## Availability and requirements

• **Project name: **CrossOver Distribution Analyzer.

• **Project home page: **http://cms.moulon.inra.fr/content/view/25/56/lang,en/

• **Operating system(s): **Gnu/Linux, MacOS X (10.4 or higher), Windows (XP or higher)

• **Programming language: **C++

• **Other requirements: **Ready-to-use executables are provided for MacOS or Windows, but installing from the sources (*e.g*. for Linux systems) needs to have Qt4, qwt and qwtplot3 d development packages installed on the system. Compiler versions: g++ v4.0.1 under MacOSX and g++ v4.4 under Windows and linux platforms. Recommanded Hardware: At least Pentium4 or equivalent, and 512 Mo RAM.

• **License: **GNU GPL

• **Any restrictions to use by non-academics: **None

Binary files for Windows and MacOS can be downloaded freely on the project home page. All sources are available under the GPL license from the same URL. The software can also be used through command lines, making it easy to perform calculations on a remote server and/or to launch analyses automatically using scripts. A detailed documentation is included in the distribution, as well as a sample data file. We request that publications using CODA refer to the present article.

## Abbreviations

CO: crossover; GUI: Graphical User Interface; SRP: stationary renewal process; BF: beam-film; BFS: beam-film sprinkling; GS: gamma sprinkling; IA: Interference Analyzer;

## Authors' contributions

All authors designed the package; OM and MF wrote the GS, BFS and statistical parts of software;

FG produced the GUI and integrated all the parts into its C++ structure. All authors wrote and approved the manuscript.

## Authors' information

The training of the authors FG, OM, and MF is respectively in computer science, physics, and genetics.
